# Lattice Boltzmann Model for Gas Flow through Tight Porous Media with Multiple Mechanisms

**DOI:** 10.3390/e21020133

**Published:** 2019-02-01

**Authors:** Junjie Ren, Qiao Zheng, Ping Guo, Chunlan Zhao

**Affiliations:** 1 School of Sciences, Southwest Petroleum University, Chengdu 610500, China; 2 State Key Laboratory of Oil and Gas Reservoir Geology and Exploitation, Southwest Petroleum University, Chengdu 610500, China

**Keywords:** Lattice Boltzmann method, tight porous media, multiple mechanisms, porous flow

## Abstract

In the development of tight gas reservoirs, gas flow through porous media usually takes place deep underground with multiple mechanisms, including gas slippage and stress sensitivity of permeability and porosity. However, little work has been done to simultaneously incorporate these mechanisms in the lattice Boltzmann model for simulating gas flow through porous media. This paper presents a lattice Boltzmann model for gas flow through porous media with a consideration of these effects. The apparent permeability and porosity are calculated based on the intrinsic permeability, intrinsic porosity, permeability modulus, porosity sensitivity exponent, and pressure. Gas flow in a two-dimensional channel filled with a homogeneous porous medium is simulated to validate the present model. Simulation results reveal that gas slippage can enhance the flow rate in tight porous media, while stress sensitivity of permeability and porosity reduces the flow rate. The simulation results of gas flow in a porous medium with different mineral components show that the gas slippage and stress sensitivity of permeability and porosity not only affect the global velocity magnitude, but also have an effect on the flow field. In addition, gas flow in a porous medium with fractures is also investigated. It is found that the fractures along the pressure-gradient direction significantly enhance the total flow rate, while the fractures perpendicular to the pressure-gradient direction have little effect on the global permeability of the porous medium. For the porous medium without fractures, the gas-slippage effect is a major influence factor on the global permeability, especially under low pressure; for the porous medium with fractures, the stress-sensitivity effect plays a more important role in gas flow.

## 1. Introduction

Gas flows in tight porous media have recently attracted much attention because they are widely applied in engineering fields, such as oil-gas field development, chemical processes, new energy development, and so on. The wide applications of gas flows in tight porous media also stimulate great interest in experimental and theoretical studies [1,2,3,4,5,6,7,8,9,10,11,12,13]. Gas flow in porous media usually involves three scales: Pore scale, representative elementary volume (REV) scale, and domain scale [14]. An REV in a porous medium is the smallest volume at which the scale characteristics of the porous flow hold, and thus the flow in a porous medium can be simulated at the REV scale without detailed information of the pore structures. Compared with pore-scale models, REV-scale models have a higher computational efficiency. Therefore, recently, many REV-scale models have been proposed in the literature, such as the Darcy model, extended Darcy models (including Brinkman-extended Darcy and Forchheimer–Darcy models), and generalized model based on the generalized Navier–Stokes equations. Because the generalized Navier–Stokes equations [15] can consider the combined influences of the fluid and solid drag forces, they have received much attention recently. In general, these REV-scale models can be solved by the conventional numerical methods, such as the finite difference method, finite volume method, and so on [16].

The lattice Boltzmann method (LBM) is considered a promising method for simulating fluid flow and has been successfully applied to the flow in tight porous media [17,18,19,20,21]. According to the spatial scale in simulations, the lattice Boltzmann (LB) model for porous flow can be classified into two categories, i.e., the pore-scale LB model and REV-scale LB model, which are used to simulate the flow in porous media at the pore and REV scales, respectively. Owing to the advantage of the LBM in the simulation of fluid dynamics, some REV-scale LB models have been proposed [22,23,24]. However, the macroscopic equations derived from these REV-scale LB models are the Darcy equation and extended Darcy equations, which cannot consider the effects of all the fluid and solid drag forces. Guo and Zhao [14] presented a generalized REV-scale LB model for incompressible flow through porous media, which can recover the generalized Navier–Stokes equations [15] in the incompressible limit. Later, Guo and Zhao [25] extended the generalized REV-scale LB model [14] to thermal flow in porous media, which can be used to simulate both the velocity and temperature fields of the porous flow. Rong et al. [26] proposed an REV-scale LB model for axisymmetric thermal flow in porous media. Recently, Chen et al. [27] proposed a generalized REV-scale LB model with the Klinkenberg’s effect based on the model proposed by Guo and Zhao [14]. It was found that the Klinkenberg’s effect plays an important role in gas flow in tight porous media, especially when the reservoir pressure decreases. 

However, in practice, gas flow through porous media in tight gas reservoirs usually takes place deep underground, where multiple mechanisms, including gas slippage and stress sensitivity of permeability and porosity, have proven to appear as the gas flows through porous media under gas-reservoir conditions [28,29,30]. To our knowledge, no work has been done to simultaneously incorporate these mechanisms in the LB model for simulating gas flow through porous media. Therefore, the aim of this paper is to propose an REV-scale LB model for gas flow through porous media with the consideration of these multiple mechanisms. The effects of these mechanisms on gas flow in tight porous media with (or without) fractures will be studied in detail.

## 2. Generalized Model for Gas Flow in Tight Porous Media

### 2.1. Generalized Navier–Stokes Equations

Owing to the nano/micron-sized porous media considered in the present work, the pressure difference in these porous media under gas-reservoir conditions is so small that the flow can be considered incompressible flow in simulations. Recently, the generalized Navier–Stokes equations proposed by Nithiarasu et al. [15] have been widely used to study the isotheral incompressible flow in porous media, which can be expressed as follows:

(1a) ∇⋅u=0,

(1b) $$\partial_{t}u+\left(u\cdot \nabla \right)\left(\frac{u}{\phi }\right)=-\frac{1}{\rho }\nabla p+\nu_{e}\nabla^{2}u+F,$$

where u is the volume-averaged velocity, t is time, ϕ is the porosity, ρ is the fluid density, p is the pressure, $\nu_{e}$ is the effective viscosity, F is the total body force, including the porous-medium resistance and other external forces expressed as follows:

(2) $$F=-\frac{\phi v}{K}u-\frac{\phi F_{\phi }}{\sqrt{K}}\left|u\right|u+\phi G,$$

where v is the shear viscosity that is not necessarily the same as $\nu_{e}$, K is the permeability, G is the external force, $F_{\phi }$ is the geometric function that can be expressed as [31]:

(3) $$F_{\phi }=\frac{1.75}{\sqrt{150\phi^{3}}}.$$

Note that the pressure, p, in Equation (1b) is the fluid pressure in pores instead of the volume-averaged pressure [15]. The first and second terms on the right side of Equation (2) are the linear and nonlinear drag forces due to the presence of the porous media, respectively. Strictly speaking, the nonlinear term in Equation (2) should be considered for tight matrix and fractures. For the low-speed flow in tight matrix, the value of $u^{2}$ is usually negligibly small, and thus the effect of the nonlinear drag force can be neglected for the sake of simplification, while for the high-speed flow in fractures, the nonlinear drag force should be considered owing to the large value of $u^{2}$ [32,33]. In this work, the nonlinear drag force is considered for tight matrix and fractures.

### 2.2. Gas Slippage Effect

Owing to the small pore size in tight porous media, the Knudsen number, Kn=λ/r, where λ represents the mean free path of the gas and r is the characteristic pore size, is usually so large that the gas-slippage effect takes place and the apparent permeability increases. It should be noted that the apparent permeability reflects the real gas-transport capacity in porous media, which is dependent on the characteristics of the porous media and fluid. The initial intrinsic permeability, which only depends on the initial porous structures, is the measured liquid permeability when the pressure is the initial reservoir pressure. The relationship between the apparent permeability and initial intrinsic permeability can be given as follows:

(4) $$K_{a}=K_{i}f_{c},$$

where $K_{a}$ is the apparent permeability, $K_{i}$ is the initial intrinsic permeability, $f_{c}=f_{p}\cdot f_{s}$ is the total correction factor, $f_{p}$ is the correction factor due to the stress-sensitivity porous structures, $f_{s}$ is the correction factor due to the gas-slippage effect.

Note that the porous media are located deep underground, and thus the pore structures are sensitive to the pore pressure (or effective stress), and the permeability and porosity are functions of the pore pressure (or effective stress). 

A detailed description of the stress sensitivity of permeability and porosity will be presented in the next section. Here, the stress-dependent measured liquid permeability, $K_{d}$, can be expressed as follows:

(5) $$K_{d}=K_{i}f_{p}.$$

Note that $K_{d}$ only depends on the stress-dependent porous structures, and is not affected by the fluid properties.

Similarly, the measured gas permeability without the stress sensitivity of permeability and porosity is given as follows:

(6) $$K_{s}=K_{i}f_{s},$$

where $K_{s}$ is the Klinkenberg’s corrected permeability without the effect of the stress-dependent porous structures.

To consider the influence of the gas-slippage effect, the correction factor, $f_{s}$, is introduced to correct the permeability. Currently, various expressions for $f_{s}$ are proposed to describe the gas-slippage effect. Klinkenberg [29] proposed a widely used expression as follows:

(7) $$f_{s}=1+\frac{b_{k}}{p},$$

where $b_{k}$ is the Klinkenberg’s slippage factor that usually depends on the porous structures. Nevertheless, the Klinkenberg’s correlation is only a first-order correlation, which may not be suitable for the tight porous media with a large Knudsen number. 

Tang et al. [34] proposed a second-order correlation, which can be considered a direct extension of the Klinkenberg’s correlation. The correction factor, $f_{s}$, presented by Tang et al. [34] is written as:

(8) $$f_{s}=1+\frac{A}{p}+\frac{B}{p^{2}},$$

where A and B are the slippage factors that depend on the mean free path, characteristic pore size, pressure, and so on.

In order to study the gas transport in all flow regimes, i.e., continuum flow (Kn≤0.001), slip flow (0.001<Kn≤0.1), transition flow (0.1<Kn≤10), and free molecular flow (Kn>10), some scholars recommended the correction factor, $f_{s}$, as follows [35]:

(9) $$f_{s}=\left[1+\beta \left(\mathrm{Kn}\right)\mathrm{Kn}\right]\left[1+\frac{4\mathrm{Kn}}{1-b_{s}\mathrm{Kn}}\right],$$

where $b_{s}$ is the slip coefficient and is equal to −1 for slip flow, β(Kn) is the rarefaction coefficient, which is given as [36]:

(10) $$\beta \left(\mathrm{Kn}\right)=\frac{1.358}{1+0.170{\mathrm{Kn}}^{-0.4348}}.$$

Equations (9) and (10) are derived from flows in a single pipe at micro and nano scales and have been widely applied to tight porous media. According to the definition of Kn, the mean free path, λ, and the characteristic pore size, r, should be determined to obtain the value of Kn. Based on the gas kinetic theory, the mean free path, λ, can be given by [37]:

(11) $$\lambda =\frac{\mu }{\rho }\sqrt{\frac{\pi }{2RT}},$$

where μ=ρν is the dynamic viscosity, R is the gas constant, T is the temperature. Following the suggestion of Ziarani and Aguilera [38], the characteristic pore size, r, can be evaluated by [39]:

(12) $$r=8.886\times 10^{-2}{\left(\frac{K_{d}}{\phi_{d}}\right)}^{0.5},$$

where $\phi_{d}$ is the stress-dependent porosity, and the units of r and $K_{d}$ are micron (μm) and millidarcy (mD), respectively. 

### 2.3. Stress Sensitivity of Permeability and Porosity

The porous media located deep underground usually suffer from overburden pressure due to the presence of overlying rocks. In the development of oil and gas reservoirs, the pore pressure decreases as the fluid in the porous media is exploited. Therefore, the effective stress, which is approximately equal to the overburden pressure minus the pore pressure, will increase. The increase of the effective stress will result in the deformation of the reservoir rocks and then reduce the permeability and porosity. This phenomenon is called stress sensitivity of permeability and porosity. Many studies [28,30] have pointed out that these stress-sensitivity characteristics in low permeable reservoirs are especially obvious, and thus the effect of the stress sensitivity on the permeability and porosity should be considered in the simulation of gas flow in tight porous media.

The relationship between the permeability and the pore pressure is usually described by [40]:

(13) $$K_{d}=K_{i}\exp\left[-\gamma \left(p_{i}-p\right)\right],$$

where $p_{i}$ is the initial reservoir pressure, γ is the permeability modulus.

The permeability varies with the porosity, which follows the following expression [40]:

(14) $$\frac{K_{d}}{K_{i}}={\left(\frac{\phi_{d}}{\phi_{i}}\right)}^{\alpha },$$

where α is the porosity sensitivity exponent, $\phi_{i}$ is the porosity under the initial reservoir pressure. 

The pore structures in real porous media are so complex that it is difficult to obtain a generalized porosity-permeability relationship. Ergun [31] proposed an expression to describe the relationship between the permeability, $K_{d}$, and porosity, $\phi_{d}$:

(15) $$K_{d}=\frac{\phi_{d}^{3}d_{p}^{2}}{150{\left(1-\phi_{d}\right)}^{2}},$$

where $d_{p}$ is the solid-particle diameter. Equation (15) can be used to simulate gas flows in porous media with an approximately uniform solid-particle diameter. Note that owing to the stress sensitivity of the reservoir rocks, the solid-particle diameter, $d_{p}$, is not a constant, but a function of the pore pressure, p. However, it is difficult to determine the relationship between $d_{p}$ and p, and there is little work to study this. In this work, we only use Equation (15) to approximatively describe the relationship between the permeability, $K_{i}$, and porosity, $\phi_{i}$, under the initial reservoir pressure, $p_{i}$, and the relationship between the permeability, $K_{d}$, and porosity, *ϕ*_d_, at arbitrary reservoir pressure, p, is defined by Equation (14). The solid-particle diameter under the initial reservoir pressure is set to be $d_{\mathrm{pi}}$. Therefore, in simulations, stress sensitivity could only affect the permeability and porosity, but not affect the solid-particle diameter and the structures of the porous media.

### 2.4. Generalized Navier–Stokes Equations for Tight Porous Media

To study gas flow through tight porous media in gas reservoirs, Equations (2) and (3) should be modified to consider the effects of the gas slippage and stress-sensitivity pore structures:

(16) $$F=-\frac{\phi_{d}v}{K_{a}}u-\frac{\phi_{d}F_{\phi }}{\sqrt{K_{a}}}\left|u\right|u+\phi_{d}G,$$

(17) $$F_{\phi }=\frac{1.75}{\sqrt{150\phi_{d}^{3}}}.$$

In summary, Equations (1), (16) and (17), together with the apparent permeability and porosity calculated by Equations (4), (9), (10), (13)–(15), compose the generalized Navier–Stokes equations for gas flow in tight porous media with multiple mechanisms.

## 3. Lattice Boltzmann Model 

There has been considerable debate in the literature about the appropriateness of the LB model with single relaxation time for simulating Darcy flows in granular porous domains and permeability estimates [41], and the LB model with multiple relaxation times is superior to the LB model with a single relaxation time in stability and accuracy. However, the LB model with a single relaxation time has a higher computational efficiency than the LB model with multiple relaxation times and can be used to accurately simulate the porous flow at the REV scale [14,25,26,27]. Therefore, to simulate the gas flow in tight porous media with multiple mechanisms, we develop an LB model with a single relaxation time based on the work of Guo and Zhao [14] for gas flow through tight porous media with multiple mechanisms, which is given as follows:

(18) $$f_{i}\left(r+c_{i}\delta_{t},t+\delta_{t}\right)-f_{i}\left(r,t\right)=-\frac{1}{\tau }\left[f_{i}\left(r,t\right)-f_{i}^{\left(\mathrm{eq}\right)}\left(r,t\right)\right]+\delta_{t}J_{i},$$

where:

(19) $$f_{i}^{\left(\mathrm{eq}\right)}=\omega_{i}\rho \left[1+\frac{c_{i}\cdot u}{c_{s}^{2}}+\frac{{\left(c_{i}\cdot u\right)}^{2}}{2\phi_{d}c_{s}^{4}}-\frac{u\cdot u}{2\phi_{d}c_{s}^{2}}\right],$$

(20) $$J_{i}=\left(1-\frac{1}{2\tau }\right)\rho \omega_{i}\left[\frac{c_{i}}{c_{s}^{2}}+\frac{\left(c_{i}\cdot u\right)c_{i}-c_{s}^{2}u}{\phi_{d}c_{s}^{4}}\right]\cdot F,$$

$f_{i}$ is the density distribution function associated with the discrete velocity, $c_{i}$, at the site, r, and time t, $f_{i}^{\left(\mathrm{eq}\right)}$ is the equilibrium distribution function, $\delta_{t}$ is the time step, τ is the dimensionless relaxation time. For the two-dimensional nine-velocity (D2Q9) model, the weight coefficients, *ω*_i_, are $\omega_{0}=4/9$, $\omega_{1–4}=1/9$, $\omega_{5–8}=1/36$; $c_{s}=c/\sqrt{3}$ is the lattice sound speed; $c_{i}$ is the discrete velocity, which is given by:

(21) $$c_{i}=\left\{ \begin{array}{cc} \left(0,0\right), & i=0,\\ \left(\cos\left[\left(i-1\right)\pi /2\right],\sin\left[\left(i-1\right)\pi /2\right]\right)c, & i=1–4,\\ \left(\cos\left[\left(2i-9\right)\pi /4\right],\sin\left[\left(2i-9\right)\pi /4\right]\right)\sqrt{2}c, & i=5–8, \end{array}\right.$$

where $c=\sqrt{3RT}$.

The density and velocity are defined, respectively:

(22a) $$\rho =\sum_{i}^{}f_{i},$$

(22b) $$\rho u=\sum_{i}^{}c_{i}f_{i}+\frac{\delta_{t}}{2}\rho F.$$

Through the Chapman–Enskog expansion, we can derive the generalized Navier–Stokes equations presented above in the limit of a small Mach number. The pressure, p, and effective viscosity, $\nu_{e}$, are defined, respectively:

(23) $$p=\rho c_{s}^{2},$$

(24) $$\nu_{e}=c_{s}^{2}\left(\tau -\frac{1}{2}\right)\delta_{t}.$$

It should be noted that F includes the velocity, u, and thus Equation (22b) is a nonlinear equation for the velocity, u. According to the suggestion proposed by Guo and Zhao [14], the velocity, **u**, can be obtained by:

(25) $$u=\frac{\upsilon }{c_{0}+\sqrt{c_{0}^{2}+c_{1}\left|\upsilon \right|}},$$

where **υ** is a temporal velocity given as:

(26) $$\rho \upsilon =\sum_{i}^{}c_{i}f_{i}+\frac{\delta_{t}}{2}\rho \phi_{d}G,$$

and the parameters, $c_{0}$ and $c_{1}$, are obtained by:

(27a) $$c_{0}=\frac{1}{2}\left(1+\phi_{d}\frac{\delta_{t}}{2}\frac{v}{K_{a}}\right),$$

(27b) $$c_{1}=\phi_{d}\frac{\delta_{t}}{2}\frac{F_{\phi }}{\sqrt{K_{a}}}.$$

The present LB model, including Equations (18)–(27), can be used to study the gas flow in the low permeable reservoirs at the REV scale with the effects of the gas slippage and stress-sensitivity pore structures.

## 4. Simulation Results and Discussions 

### 4.1. Flow in a Channel Filled with a Porous Medium

First, gas flow in a channel filled with a homogeneous porous medium is simulated to validate the present LB model. The flow is driven by pressure difference, Δp, at the inlet and outlet, which can be used to calculate the external force by G=Δp/(ρL) with L representing the length of the channel. The streamwise velocity, $u_{x}$ (i.e., x-direction velocity), can be described by the following equation [14]:

(28) $$\frac{v_{e}}{\phi_{d}}\frac{\partial^{2}u_{x}}{\partial y^{2}}+G-\frac{v}{K_{a}}u_{x}-\frac{F_{\phi }}{\sqrt{K_{a}}}u_{x}^{2}=0.$$

with $u_{x}\left(x,0\right)=u_{x}\left(x,H\right)=0$, where H is the height of the channel. Owing to the extremely low velocity in tight porous media without fractures, the nonlinear inertial effect (i.e., the fourth term on the left-hand side of Equation (28)) can be neglected. Therefore, Equation (28) is simplified as:

(29) $$\frac{v_{e}}{\phi_{d}}\frac{\partial^{2}u_{x}}{\partial y^{2}}+G-\frac{v}{K_{a}}u_{x}=0.$$

The analytical solution of Equation (29) is as follows:

(30) $$u_{x}=\frac{GK_{a}}{v}\left(1-\frac{\cosh\left[A\left(y-H/2\right)\right]}{\cosh\left(AH/2\right)}\right),$$

where $A=\sqrt{v\phi_{d}/K_{a}v_{e}}$. Note that if some fractures exist in the tight porous media, the nonlinear inertial effect needs to be considered in simulations because of the large flow rate in the fractures. In the following simulations, the effective viscosity, $\nu_{e}$, is assumed to equal the shear viscosity, v. The initial pressure, $p_{i}$, porosity sensitivity exponent, α, initial solid-particle diameter, $d_{\mathrm{pi}}$, temperature, *T*, and gas constant, R, are set as 30 MPa, 3, 50 nm, 333.15 K, and 519.6545 J/(K·kg), respectively. The dynamic viscosity, *μ*, is calculated by the method proposed by Dempsey [42].

**Figure 1:**
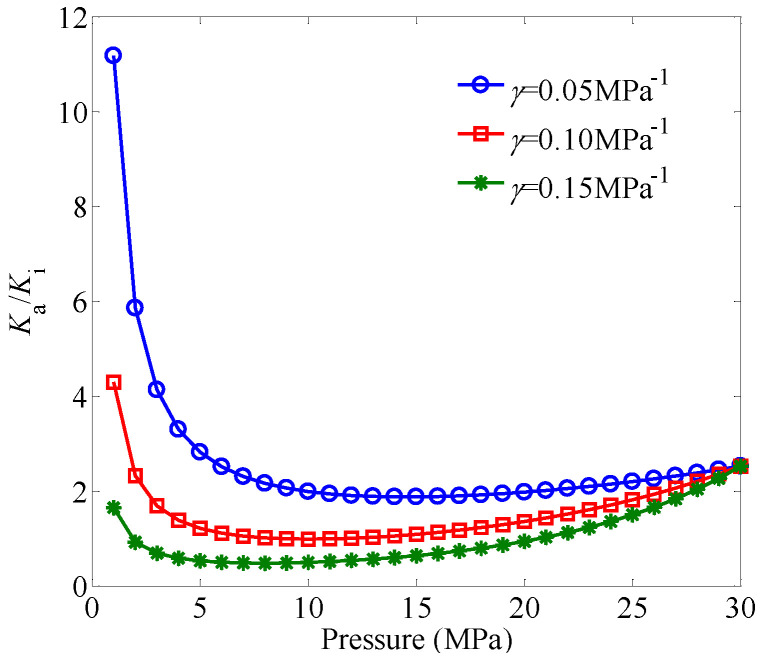
The effect of the permeability modulus on $K_{a}/K_{i}$ under different pressures. The pressure varies from 1 to 30 MPa, and the initial porosity is set as $\phi_{i}=0.1$.

Figure 1 shows the effect of the permeability modulus on *K*_a_/*K*_i_ under different pressures. It can be seen that *K*_a_/*K*_i_ first decreases as the pressure decreases from 30 MPa, and then *K*_a_/*K*_i_ increases as the pressure continues to decrease, which results from the combined influences of the gas slippage and stress-sensitivity pore structures. With the decrease of the pressure, the gas-slippage effect makes the permeability increase, while the stress-sensitivity effect makes the permeability decrease. The stress-sensitivity effect plays a more important role in gas transport with the increase of the permeability modulus. For a fixed value of the permeability modulus, the gas-slippage effect becomes gradually significant as the pressure decreases.

**Figure 2:**
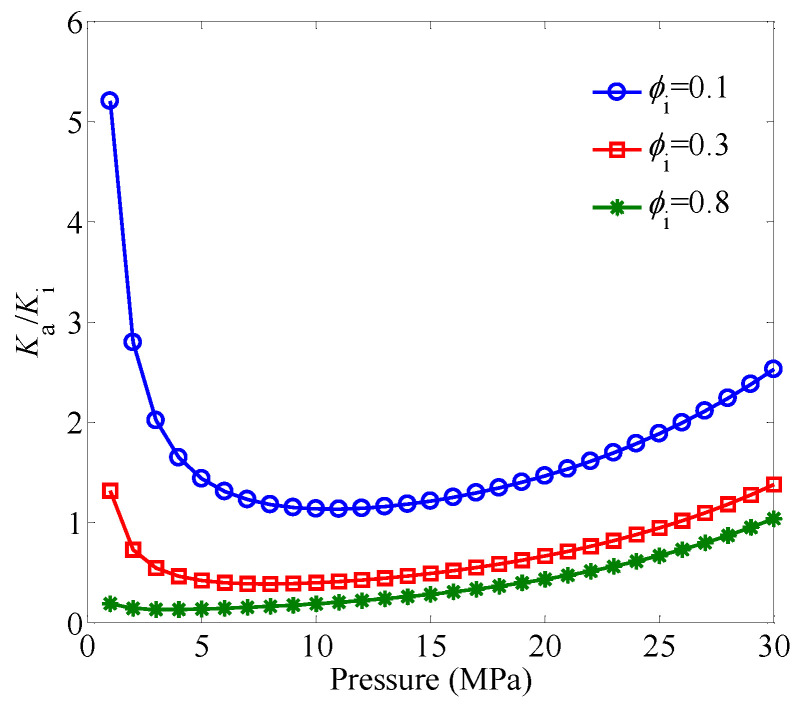
The effect of the initial porosity on $K_{a}/K_{i}$ under different pressures. The pressure varies from 1 to 30 MPa, and the permeability modulus is set as $\gamma =0.09{\;\mathrm{MPa}}^{-1}$.

Figure 2 shows the effect of the initial porosity on $K_{a}/K_{i}$ under different pressures. It is clear that the gas-slippage effect becomes more obvious with the decrease of the initial porosity. For a fixed value of the initial porosity, the gas-slippage effect increases with the decrease of the pressure.

**Figure 3:**
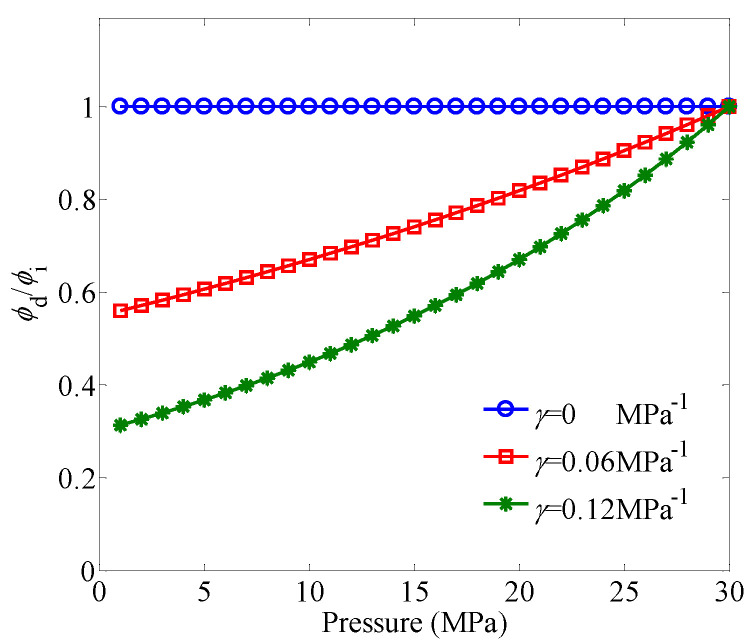
The effect of the permeability modulus on *ϕ*_d_/*ϕ*_i_ under different pressures. The pressure varies from 1 to 30 MPa, and the initial porosity is set as $\phi_{i}=0.1$.

Figure 3 shows the effect of the permeability modulus on $\phi_{d}/\phi_{i}$ under different pressures. For a given pressure, the value of $\phi_{d}/\phi_{i}$ decreases with the increase of the permeability modulus. When γ=0, the stress-sensitivity effect is neglected, and thus the porosity remains constant under different pressures. When the value of γ is small (e.g., $\gamma \leq 0.06{\;\mathrm{MPa}}^{-1}$), the porosity decreases linearly as the pressure decreases. However, with the increase of the value of γ, a nonlinear relationship between the porosity and pressure will appear.

In the simulations, the present LB model in lattice units is implemented, and then the simulation results are converted from lattice units to physical units [43]. Both the length and height of the channel are set as 1 μm (i.e., L×H=1 μm×1 μm), the initial porosity, $\phi_{i}$, is fixed at 0.3, and the pressure gradient between the inlet and outlet is set to be 1.0MPa/m. The lattice size is chosen as 200×200. The dimensionless relaxation time, τ, is set as 1.0. Periodic boundary conditions are applied to the inlet and outlet, and the nonequilibrium extrapolation conditions [44] are applied to the top and bottom walls. The initial velocities ($u_{x}$ and $u_{y}$) are set to be 0, and the initial density, ρ, is set as 1.0. The convergence criterion is given as follows:

(31) $$\sqrt{\frac{\sum_{i,j}\left\{{\left[u_{x}\left(i,j,t+100\delta_{t}\right)-u_{x}\left(i,j,t\right)\right]}^{2}+{\left[u_{y}\left(i,j,t+100\delta_{t}\right)-u_{y}\left(i,j,t\right)\right]}^{2}\right\}}{\sum_{i,j}\left[u_{x}{\left(i,j,t+100\delta_{t}\right)}^{2}+u_{y}{\left(i,j,t+100\delta_{t}\right)}^{2}\right]}}<10^{-6}.$$

**Figure 4:**
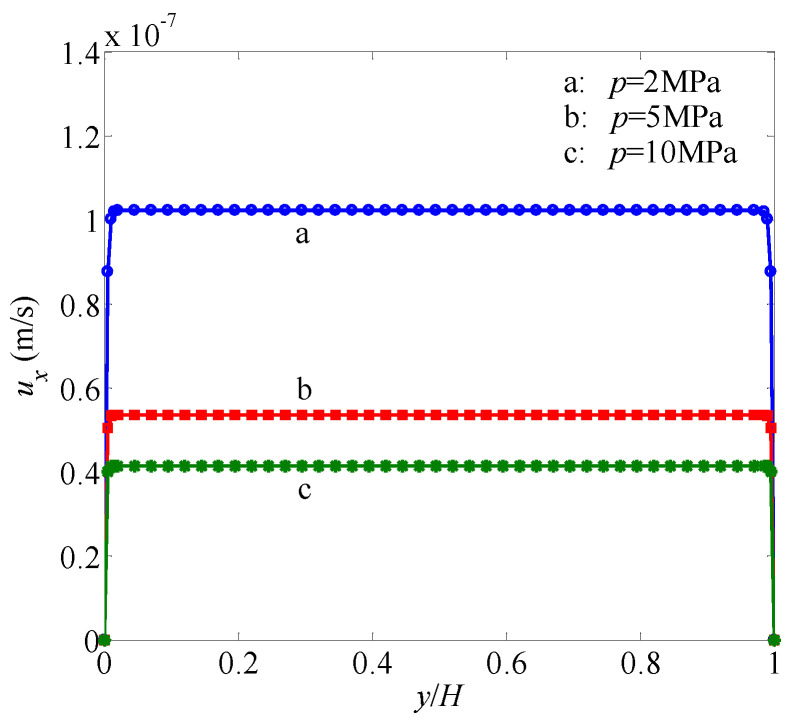
Velocity profiles in a channel filled with a porous medium under different pressures. Solid lines represent the analytical solutions and symbols represent the simulation results by the present model. The permeability modulus is set as $\gamma =0.06{\;\mathrm{MPa}}^{-1}$.

**Figure 5:**
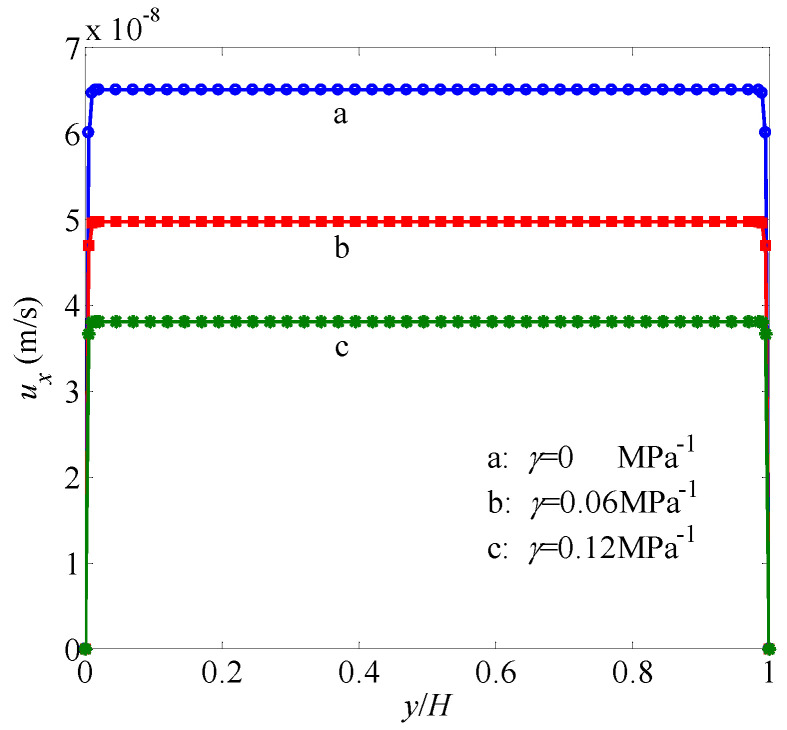
Velocity profiles in a channel filled with a porous medium under different permeability moduli. Solid lines represent the analytical solutions and symbols represent the simulation results by the present model. The pressure is set as p=25 MPa.

The simulation results are shown in Figure 4 and Figure 5, from which it is seen that the simulation results by the present LB model are in excellent agreement with the analytic solutions in all cases with different permeability moduli and pore pressures. Therefore, the present LB model has the capability to simulate gas flow through tight porous media with multiple mechanisms, including gas slippage and stress sensitivity of permeability and porosity.

### 4.2. Flow in Tight Porous Media without Fractures

**Figure 6:**
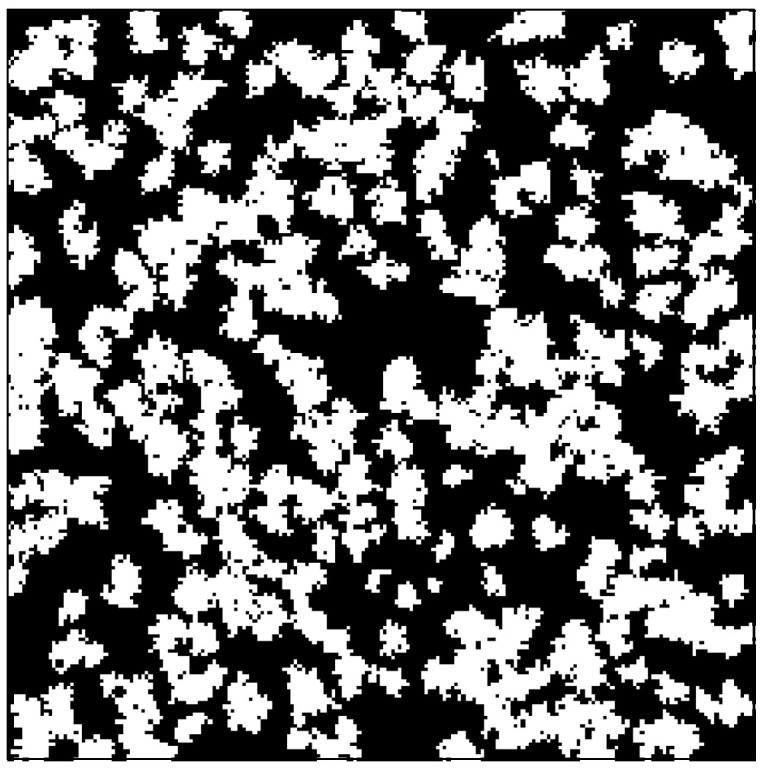
Structure of a heterogeneous porous medium with two mineral components: organic material with relatively lower permeability and porosity (white), and inorganic material with relatively higher permeability and porosity (black). The randomly distributed organic material is generated by the QSGS method. Volume fractions of the inorganic material and organic material are 0.58 and 0.42, respectively.

In practice, the real porous media are not homogeneous systems with uniform permeability and porosity, but heterogeneous systems that consist of different mineral components. Taking shale matrix as an example, shale matrix mainly consists of organic material and inorganic material. In general, the pores in shale matrix are nanoscale pores, and thus gas transport in both organic and inorganic materials is extremely slow. Compared with the inorganic material, organic material has a relatively lower permeability and porosity. Therefore, it is essential to study gas flow in porous media with different mineral components. In this section, the structure of a heterogeneous porous medium with two mineral components is considered, which is shown in Figure 6. The randomly distributed organic material is generated by the quartet structure generation set (QSGS) method [45]. The volume fractions of the inorganic material and organic material are 0.58 and 0.42, respectively. The domain size of the porous medium is L×H=2 μm×2 μm. The initial porosities of inorganic material and organic material are 0.3 and 0.1, respectively. The permeability moduli of inorganic material and organic material are $0.06\;{\mathrm{MPa}}^{-1}$ and $0.12\;{\mathrm{MPa}}^{-1}$, respectively. Periodic boundary conditions are applied to all the boundaries. The pressure gradient between the inlet and outlet is set to be 1.0 MPa/m. The dimensionless relaxation time, τ, is set as 1.0. The initial conditions and convergence criterion are the same as that in Section 4.1. The values of parameters in physical units and lattice units for simulations of gas flow in tight porous media without fractures are listed in Table 1 and Table 2, respectively.

**Figure 7:**
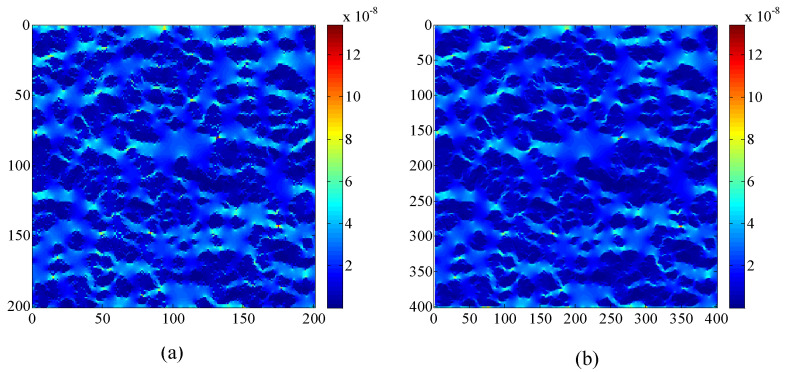
Velocity magnitude distributions in the porous medium based on different grid numbers. The pressure is set as p=10 MPa. (**a**) 200 × 200; (**b**) 400 × 400.

**Figure 8:**
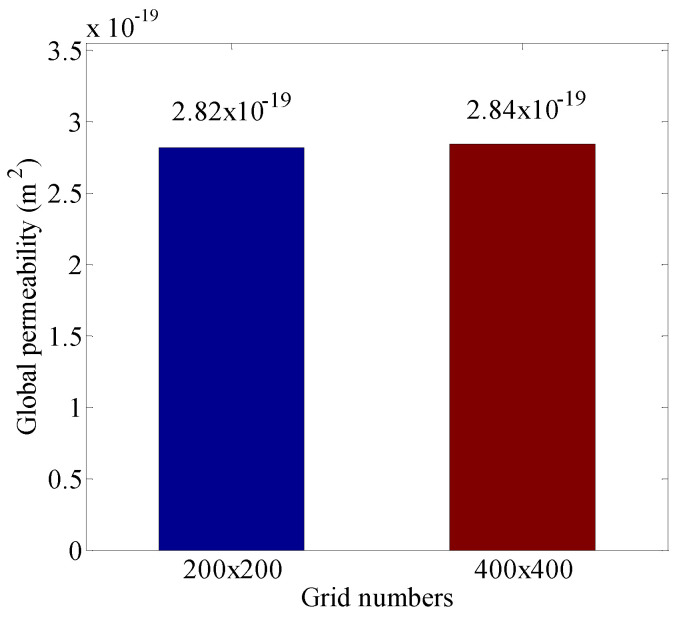
Global permeability based on different grid numbers. The pressure is set as p=10 MPa.

The grid dependency for the simulation results is tested based on the structure of the porous medium shown in Figure 6. The two different grid sizes, namely 200 × 200 and, 400 × 400 are employed to simulate the gas flow. The effect of the grid number on the velocity magnitude distributions in the porous medium is shown in Figure 7. The velocity magnitude, |u|, is defined as $\left|u\right|=\sqrt{u_{x}^{2}+u_{y}^{2}}$. There is no obvious difference between the velocity magnitude distributions based on the two grid system. The global permeability is introduced to quantify the seepage capability of the porous medium. Note that the global permeability defined by Chen et al. [27] is dependent on the flow field in the entire domain rather than the local characteristic of a porous medium. The definition of the global permeability is given as follows:

(32) $$K_{g}=\frac{v\int_{0}^{H}\left|u\left(L,y\right)\right|dy}{H\cdot G},$$

where $K_{g}$ is the global permeability. It is seen from Figure 8 that no obvious change of the global permeability is observed when the grid is refined, and thus the 200×200 lattice is sufficient to obtain grid-independent results.

**Figure 9:**
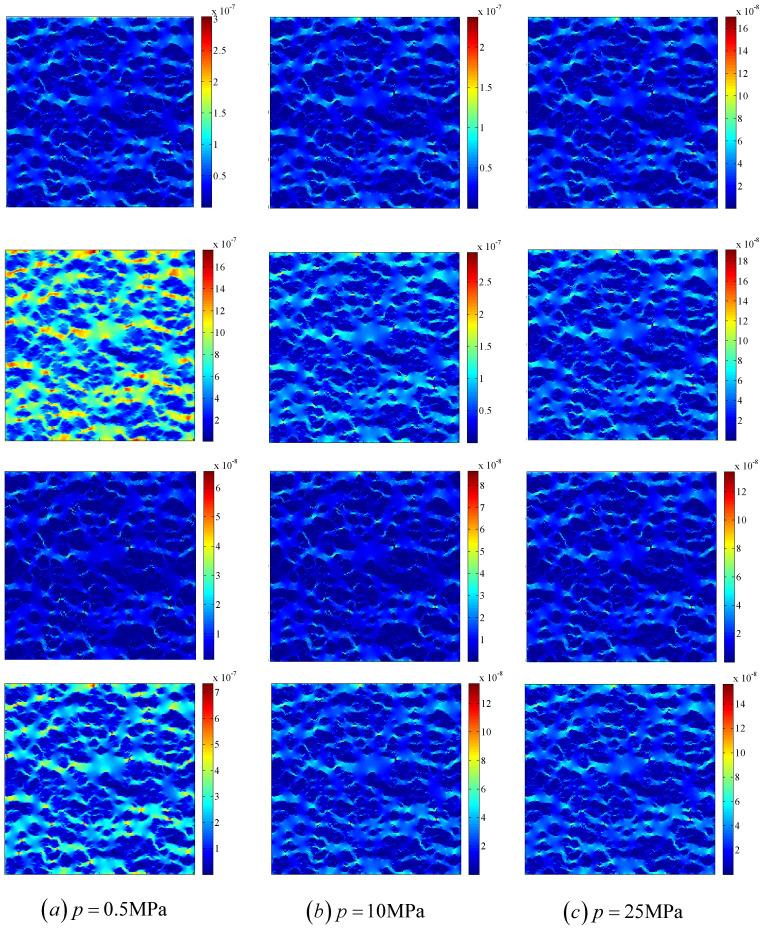
The effects of the gas slippage and stress-sensitivity pore structures on velocity magnitude distributions in the porous medium. (**a**) p=0.5 MPa, (**b**) p=10 MPa, (**c**) p=25 MPa. The first row: without the effects of gas slippage and stress-sensitivity pore structures; the second row: with only the effect of gas slippage; the third row: with only the effect of stress-sensitivity pore structures; the fourth row: with gas slippage and stress-sensitivity pore structures.

**Figure 10:**
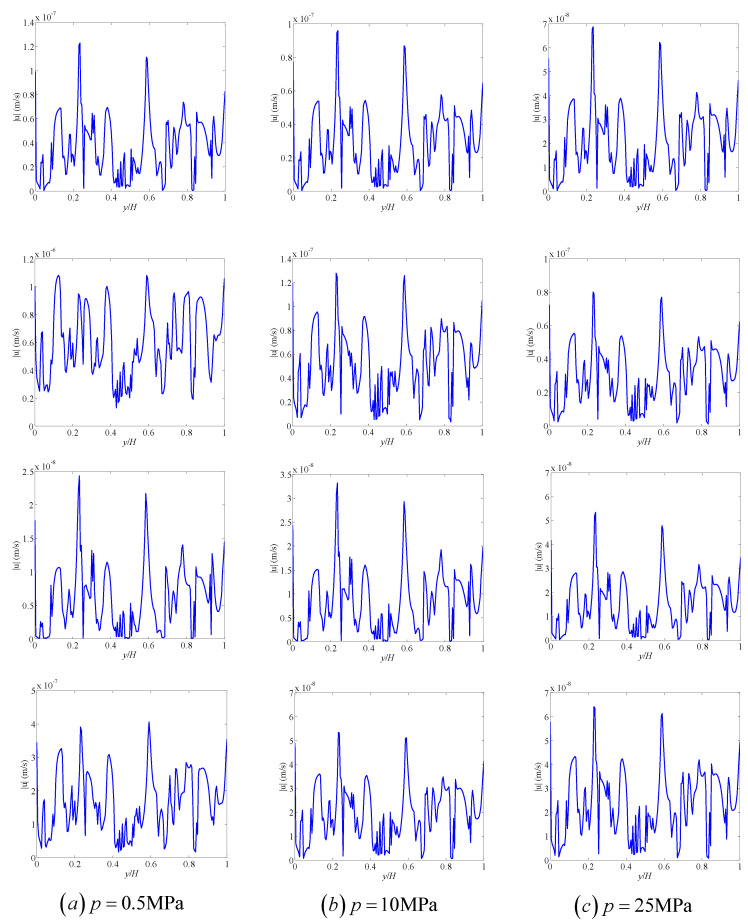
The effects of the gas slippage and stress-sensitivity pore structures on velocity magnitude profiles at the outlet of the porous medium. (**a**) p=0.5 MPa, (**b**) p=10 MPa, (**c**) p=25 MPa. The first row: without the effects of gas slippage and stress-sensitivity pore structures; the second row: with only the effect of gas slippage; the third row: with only the effect of stress-sensitivity pore structures; the fourth row: with gas slippage and stress-sensitivity pore structures.

Figure 9 shows the effects of the gas slippage and stress-sensitivity pore structures on velocity magnitude distributions in the porous medium. The gas-slippage effect enhances the global velocity magnitude, while the stress-sensitivity effect reduces the global velocity magnitude as shown in Figure 9. It is seen that under a fixed pressure, the velocity magnitude distributions are different for the cases with and without the gas-slippage and stress-sensitivity effects, which is because these influencing factors have different effects on gas transport in different materials. Figure 10 shows the effects of the gas slippage and stress-sensitivity pore structures on the velocity magnitude profiles at the outlet of the porous medium. It is clear that the velocity magnitude profiles at the outlet are also different for the cases with and without the gas-slippage and stress-sensitivity effects. Therefore, multiple mechanisms, including gas slippage and stress sensitivity of permeability and porosity, affect not only the global velocity magnitude, but also the flow field. To obtain a correct prediction of gas flow in porous media, these mechanisms should be considered in simulations.

**Figure 11:**
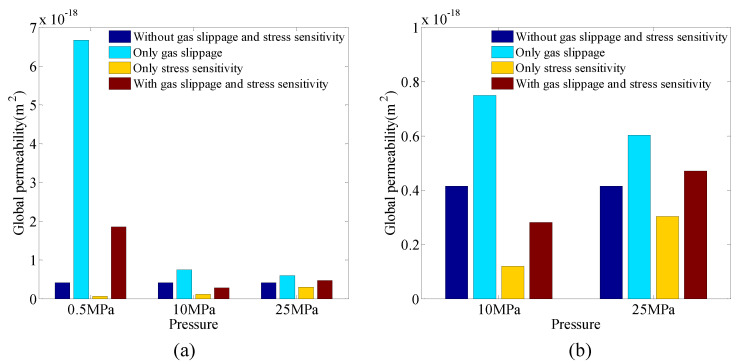
The effects of the gas slippage and stress-sensitivity pore structures on the global permeability under different pressures. (**b**) is a subfigure of (**a**).

We will focus on the effects of the multiple mechanisms on the global permeability. Figure 11 shows the effects of the gas slippage and stress-sensitivity pore structures on global permeability under different pressures. It is observed that the gas slippage and stress-sensitivity pore structures significantly affect the global permeability. The gas-slippage effect increases the global permeability and becomes stronger with a decrease of the pressure. The stress-sensitivity effect decreases the global permeability as the pressure is reduced. In general, the global permeability of a porous medium deep underground is affected by the combined influences of the gas slippage and stress sensitivity. The quantitative comparison between the global permeability with and without the effects of the gas slippage and stress-sensitivity pore structures is listed in Table 3, from which it can be seen that compared with the stress-sensitivity effect, the gas-slippage effect becomes more significant in matrix, especially under low pressure.

### 4.3. Flow in Tight Porous Media with Fractures

**Figure 12:**
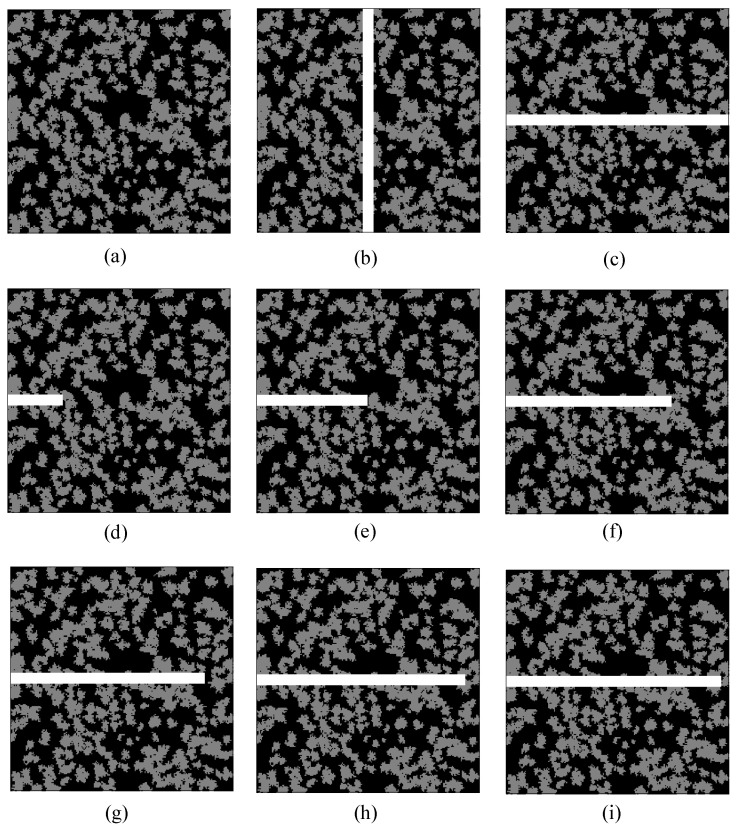
Structures of a heterogeneous porous medium with no fracture (**a**), a completely penetrated fracture along the vertical centerline (**b**), a completely penetrated fracture along the horizontal centerline (**c**), and partly penetrated fractures along the horizontal centerline with the penetration ratio being 1/4 (**d**), 1/2 (**e**), 3/4 (**f**), 7/8 (**g**), 15/16 (**h**), and 31/32 (**i**). White represents the fractures, black represents the inorganic matrix, and gray represents the organic matrix.

In general, natural fractures may exist in a porous medium. Compared with the matrix with organic and inorganic materials, natural fractures can significantly enhance the gas-transport capability of a porous medium. In this section, gas flow in tight porous media with fractures will be studied. The effects of the fracture distributions, gas slippage, and stress sensitivity on gas transport are analyzed in detail. The distributions of the organic and inorganic materials in a porous medium are the same as that in Figure 6. The fractures with different lengths and directions are embedded in the porous medium as shown in Figure 12. The domain size of the porous medium is 20 μm×20 μm, and the width of the fracture is set as 1 μm. Generally, gas flow in fractures can be approximately considered as the seepage in a porous medium with extremely high porosity and permeability. The initial porosity and permeability modulus of the fractures are 0.99 and $0.03{\;\mathrm{MPa}}^{-1}$, respectively. Periodic boundary conditions are applied to all the boundaries. The pressure gradient between the inlet and outlet is set to be 1.0 MPa/m. The dimensionless relaxation time, τ, is set as 1.0. The initial conditions and convergence criterion are the same as that in Section 4.1. The values of parameters in physical units and lattice units for simulations of gas flow in tight porous media with fractures are listed in Table 4.

**Figure 13:**
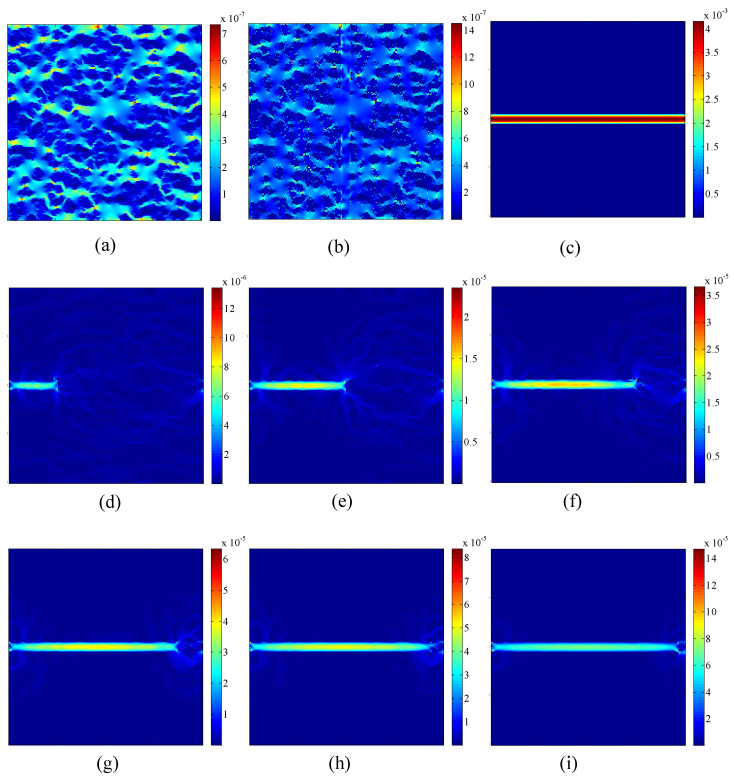
The effects of the fracture distributions on velocity magnitude distributions in a porous medium under the pressure of 0.5 MPa. (**a**–**i**) are the simulation results based on the structures of the porous medium shown in Figure 12a–i, respectively.

Figure 13 shows the effects of the fracture distributions on velocity magnitude distributions in a porous medium. It is seen that the fracture distributions have an important effect on the flow field. When the fracture is perpendicular to the pressure-gradient direction, there is a small change of the flow field compared with a porous medium without fractures. However, when the fracture is along the pressure-gradient direction, a large change of the flow field will appear. Furthermore, the average velocity in the fracture along the pressure-gradient direction increases observably compared with that in the fracture perpendicular to the pressure-gradient direction. The average velocity in the fracture along the pressure-gradient direction increases as the length of the fracture increases. The comparison between the global permeability of a porous medium with and without fractures under the pressure of 0.5 MPa is shown in Table 5. It is observed that the fracture along the pressure-gradient direction enhances the global permeability more significantly compared with the fracture perpendicular to the pressure-gradient direction. For the fracture along the pressure-gradient direction, the penetration ratio of the fracture in the porous medium is also an important factor on the global permeability. It is found that the global permeability with completely penetrated fracture is obviously higher than that with partly penetrated fracture, and the global permeability increases when the penetration ratio increases.

The effects of the gas slippage and stress-sensitivity pore structures on global permeability of a porous medium with fractures are studied. The quantitative results are presented in Table 6, from which it is seen that the gas-slippage effect has little effect on the global permeability, while the stress-sensitivity effect significantly affects it. According to the comparison between Table 3 and Table 6, it is found that for the porous medium without fractures, the gas-slippage effect is a major influence factor on the global permeability, especially under low pressure; for the porous medium with fractures, the stress-sensitivity effect plays a more important role in gas flow.

## 5. Conclusions

In this paper, the generalized Navier–Stokes equations were proposed for simulating gas flow through porous media with the effects of gas slippage and stress-sensitivity porous structures. The apparent permeability and porosity were obtained based on the intrinsic permeability, intrinsic porosity, permeability modulus, porosity sensitivity exponent, and pressure. An LB model was developed to solve the generalized Navier–Stokes equations. Gas flow in a two-dimensional channel filled with a homogeneous porous medium was simulated to validate the present LB model. The simulation of gas flow in a porous medium with different mineral components was carried out. The simulation results showed that the gas-slippage effect increases the global permeability of the porous medium and becomes stronger with the decrease of the pressure, while the stress-sensitivity effect decreases the global permeability as the pressure decreases. Furthermore, it was found that these effect factors affect not only the global permeability, but also the flow field. Gas flow in a porous medium with fractures was also investigated. It was found that the fractures along the pressure-gradient direction significantly enhance the total flow rate of the porous medium, while the fractures perpendicular to the pressure-gradient direction have little effect on the global permeability. For the porous medium without fractures, the gas-slippage effect is a major influence factor on the global permeability, especially under low pressure; for the porous medium with fractures, the stress-sensitivity plays a more important role in gas flow.

## Figures and Tables

**Table 1:** Basic parameters in physical units used for simulations of gas flow in tight porous media without fractures.

Parameter	0.5 MPa	10 MPa	25 MPa
L (m)	$2\times 10^{-6}$	$2\times 10^{-6}$	$2\times 10^{-6}$
H (m)	$2\times 10^{-6}$	$2\times 10^{-6}$	$2\times 10^{-6}$
$G\;\left(\mathrm{Pa}\cdot m^{2}/\mathrm{kg}\right)$	$3.4625\times 10^{5}$	$1.7312\times 10^{4}$	$6.9249\times 10^{3}$
$v\;\left(\mathrm{Pa}\cdot s\cdot m^{3}/\mathrm{kg}\right)$	$3.8651\times 10^{-6}$	$2.4740\times 10^{-7}$	$1.3805\times 10^{-7}$
Inorganic material $K_{a}\;\left(m^{2}\right)$	$4.1931\times 10^{-18}$	$5.9209\times 10^{-19}$	$9.9148\times 10^{-19}$
Inorganic material $K_{d}\;\left(m^{2}\right)$	$1.5643\times 10^{-19}$	$2.7661\times 10^{-19}$	$6.8034\times 10^{-19}$
Inorganic material $K_{i}\;\left(m^{2}\right)$	$9.1837\times 10^{-19}$	$9.1837\times 10^{-19}$	$9.1837\times 10^{-19}$
Inorganic material $\phi_{d}$	0.1663	0.2011	0.2715
Inorganic material $\phi_{i}$	0.3	0.3	0.3
Organic material $K_{a}\;\left(m^{2}\right)$	$1.1587\times 10^{-19}$	$1.5430\times 10^{-20}$	$3.4592\times 10^{-20}$
Organic material $K_{d}\;\left(m^{2}\right)$	$5.9698\times 10^{-22}$	$1.8666\times 10^{-21}$	$1.1292\times 10^{-20}$
Organic material $K_{i}\;\left(m^{2}\right)$	$2.0576\times 10^{-20}$	$2.0576\times 10^{-20}$	$2.0576\times 10^{-20}$
Organic material $\phi_{d}$	0.0307	0.0449	0.0819
Organic material $\phi_{i}$	0.1	0.1	0.1

**Table 2:** Basic parameters in lattice units used for simulations of gas flow in tight porous media without fractures.

Parameter	0.5 MPa	10 MPa	25 MPa
L (lattice unit)	2	2	2
H (lattice unit)	2	2	2
G (lattice unit)	$2.3178\times 10^{-14}$	$2.8284\times 10^{-13}$	$3.6310\times 10^{-13}$
v (lattice unit)	$1.0\times 10^{-6}$	$1.0\times 10^{-6}$	$1.0\times 10^{-6}$
Inorganic material $K_{a}\;\left(\mathrm{lattice}\;\mathrm{unit}\right)$	$4.1931\times 10^{-6}$	$5.9209\times 10^{-7}$	$9.9148\times 10^{-7}$
Inorganic material $K_{d}\;\left(\mathrm{lattice}\;\mathrm{unit}\right)$	$1.5643\times 10^{-7}$	$2.7661\times 10^{-7}$	$6.8034\times 10^{-7}$
Inorganic material $K_{i}\;\left(\mathrm{lattice}\;\mathrm{unit}\right)$	$9.1837\times 10^{-7}$	$9.1837\times 10^{-7}$	$9.1837\times 10^{-7}$
Inorganic material $\phi_{d}$	0.1663	0.2011	0.2715
Inorganic material $\phi_{i}$	0.3	0.3	0.3
Organic material $K_{a}\;\left(\mathrm{lattice}\;\mathrm{unit}\right)$	$1.1587\times 10^{-7}$	$1.5430\times 10^{-8}$	$3.4592\times 10^{-8}$
Organic material $K_{d}\;\left(\mathrm{lattice}\;\mathrm{unit}\right)$	$5.9698\times 10^{-10}$	$1.8666\times 10^{-9}$	$1.1292\times 10^{-8}$
Organic material $K_{i}\;\left(\mathrm{lattice}\;\mathrm{unit}\right)$	$2.0576\times 10^{-8}$	$2.0576\times 10^{-8}$	$2.0576\times 10^{-8}$
Organic material $\phi_{d}$	0.0307	0.0449	0.0819
Organic material $\phi_{i}$	0.1	0.1	0.1

**Table 3:** Comparison between the global permeability with and without the effects of the gas slippage and stress-sensitivity pore structures in a porous medium without fractures. The variable with the subscript, g, represents the corresponding global permeability.

Pressure	$K_{\mathrm{gs}}/K_{\mathrm{gi}}$	$K_{\mathrm{gd}}/K_{\mathrm{gi}}$	$K_{\mathrm{ga}}/K_{\mathrm{gi}}$
0.5 MPa	16.0734	0.1621	4.4781
10 MPa	1.8065	0.2897	0.6788
25 MPa	1.4522	0.7319	1.1345

**Table 4:** Basic parameters in physical units and lattice units used for simulations of gas flow in tight porous media with fractures under the pressure of 0.5 MPa.

Parameter	Physical Unit	Lattice Unit
L	$20\times 10^{-6}\;\left(m\right)$	20
H	$20\times 10^{-6}\left(m\right)$	20
G	$3.4625\times 10^{5}\;\left(\mathrm{Pa}\cdot m^{2}/\mathrm{kg}\right)$	$2.3178\times 10^{-14}$
v	$3.8651\times 10^{-6}\;\left(\mathrm{Pa}\cdot s\cdot m^{3}/\mathrm{kg}\right)$	$1.0\times 10^{-6}$
Inorganic material $K_{a}$	$4.1931\times 10^{-18}\;\left(m^{2}\right)$	$4.1931\times 10^{-6}$
Inorganic material $K_{d}$	$1.5643\times 10^{-19}\;\left(m^{2}\right)$	$1.5643\times 10^{-7}$
Inorganic material $K_{i}$	$9.1837\times 10^{-19}\;\left(m^{2}\right)$	$9.1837\times 10^{-7}$
Inorganic material $\phi_{d}$	0.1663	0.1663
Inorganic material $\phi_{i}$	0.3	0.3
Organic material $K_{a}$	$1.1587\times 10^{-19}\;\left(m^{2}\right)$	$1.1587\times 10^{-7}$
Organic material $K_{d}$	$5.9698\times 10^{-22}\;\left(m^{2}\right)$	$5.9698\times 10^{-10}$
Organic material $K_{i}$	$2.0576\times 10^{-20}\;\left(m^{2}\right)$	$2.0576\times 10^{-8}$
Organic material $\phi_{d}$	0.0307	0.0307
Organic material $\phi_{i}$	0.1	0.1
Natural fracture $K_{a}$	$7.0996\times 10^{-14}\;\left(m^{2}\right)$	$7.0996\times 10^{-2}$
Natural fracture $K_{d}$	$6.6743\times 10^{-14}\;\left(m^{2}\right)$	$6.6743\times 10^{-2}$
Natural fracture $K_{i}$	$1.6172\times 10^{-13}\;\left(m^{2}\right)$	$1.6172\times 10^{-1}$
Natural fracture $\phi_{d}$	0.7371	0.7371
Natural fracture $\phi_{i}$	0.99	0.99

**Table 5:** Comparison between the global permeability of a porous medium with and without the fractures under the pressure of 0.5 MPa.

Structures of a Porous Medium with Fracture	The Ratio of Global Permeability with Fracture and Global Permeability without Fracture
Figure 12b	1.4037
Figure 12c	938.2724
Figure 12d	2.6933
Figure 12e	5.0843
Figure 12f	7.6190
Figure 12g	11.1979
Figure 12h	13.4764
Figure 12i	18.1392

**Table 6:** Comparison between the global permeability with and without the effects of the gas slippage and stress-sensitivity pore structures in a porous medium with fractures. The structure of the porous medium is shown in Figure 12c. The variable with the subscript, g, represents the corresponding global permeability.

Pressure	$K_{\mathrm{gs}}/K_{\mathrm{gi}}$	$K_{\mathrm{gd}}/K_{\mathrm{gi}}$	$K_{\mathrm{ga}}/K_{\mathrm{gi}}$
0.5 MPa	1.0226	0.5594	0.5797
10 MPa	1.0014	0.6807	0.6820
25 MPa	1.0008	0.9116	0.9123
